# Genetic Basis of Inherited Macular Dystrophies and Implications for Stem Cell Therapy

**DOI:** 10.1002/stem.159

**Published:** 2009-06-23

**Authors:** Carla B Mellough, David HW Steel, Majlinda Lako

**Affiliations:** a Institute of Human Genetics andInternational Centre for LifeNewcastle Upon Tyne, United Kingdom; b North East Stem Cell Institute, Newcastle University, International Centre for LifeNewcastle Upon Tyne, United Kingdom; c Sunderland Eye InfirmaryQueen Alexandra Road, Sunderland, Tyne and Wear, United Kingdom

**Keywords:** Hereditary disease, Stem cells, gene therapy, Macular degeneration, Retinal photoreceptors, Cell therapy, Induced pluripotency, Embryonic stem cells

## Abstract

Untreatable hereditary macular dystrophy (HMD) presents a major burden to society in terms of the resulting patient disability and the cost to the healthcare provision system. HMD results in central vision loss in humans sufficiently severe for blind registration, and key issues in the development of therapeutic strategies to target these conditions are greater understanding of the causes of photoreceptor loss and the development of restorative procedures. More effective and precise analytical techniques coupled to the development of transgenic models of disease have led to a prolific growth in the identification and our understanding of the genetic mutations that underly HMD. Recent successes in driving differentiation of pluripotent cells towards specific somatic lineages have led to the development of more efficient protocols that can yield enriched populations of a desired phenotype. Retinal pigmented epithelial cells and photoreceptors derived from these are some of the most promising cells that may soon be used in the treatment of specific HMD, especially since rapid developments in the field of induced pluripotency have now set the stage for the production of patient-derived stem cells that overcome the ethical and methodological issues surrounding the use of embryonic derivatives. In this review we highlight a selection of HMD which appear suitable candidates for combinatorial restorative therapy, focusing specifically on where those photoreceptor loss occurs. This technology, along with increased genetic screening, opens up an entirely new pathway to restore vision in patients affected by HMD.

## INTRODUCTION

**Figure 1:**
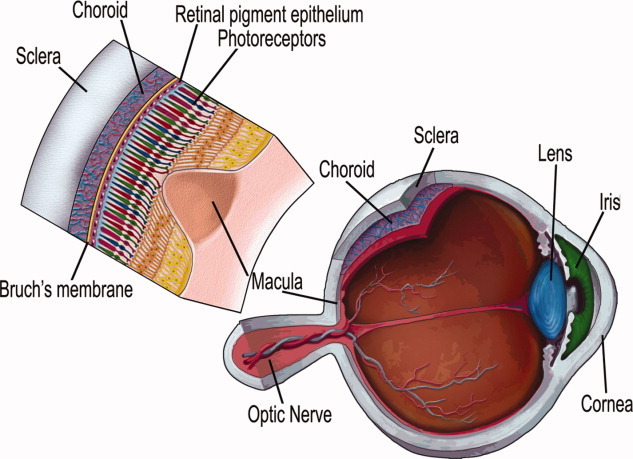
A schematic of the human eye showing the location of the macula, and an enlarged section of the retina at this region showing the organization of the retinal layers and the close relationship of the photoreceptors with the retinal pigment epithelium, Bruch's membrane, and the choriocapillaris of the choroid.

Numerous hereditary retinal disorders (HRD) that affect central visual function in humans have been identified. The specialized central region of the retina, the macula, is responsible for central visual acuity and has distinct anatomical and physiological properties. The fovea at the epicentre of the macula contains the highest density of retinal cone photoreceptors and receives its blood supply entirely from the choriocapillaris complex of the choroid. Derived from the optic neuroepithelium during eye development, the photoreceptors share a common embryological origin with and are supported by the retinal pigmented epithelium (RPE) [1,2], which is separated from the choriocapillaris by Bruch's membrane, a multilayered basement membrane (Fig. 1). Photoreceptors, RPE, and the choriocapillaris are interdependent on each other; photoreceptor dysfunction and degeneration can occur secondary to RPE pathology (e.g., in Best's macular dystrophy) or be a primary event such as is the case in Stargardt disease (STGD), where RPE pathophysiology resulting from photoreceptor malfunction leads to photoreceptor depletion. Choriocapillaris atrophy and histopathology can be observed after RPE degeneration in various fundus disorders [3,4]. Such features are characteristic of a heterogeneous subgroup of progressive HRD affecting the macula, which can cause profound central visual loss sufficiently severe enough for blind registration. Blindness substantially impairs an individual's quality of life [5–8] and places a large burden on healthcare and support services [9]. There is currently no cure for the underlying causes of any of the macular dystrophies, and available treatments are largely palliative.

Much effort has been invested in the development of protective and cell-based therapies for HRD. Some success has been achieved in prolonging the survival of photoreceptors by intraocular application of growth or antiapoptotic factors in humans [10]; however, even with repeated application this approach typically slows the progression but fails to arrest the degenerative process [11]. The transfer of genes encoding for these factors into the eye offers additional protection by modifying the physiology of affected cells; however, this approach is ineffective in cells that are dysfunctional due to an underlying genetic mutation. RPE transplantation prevents photoreceptor degeneration in models of RPE dystrophy [12,13], but not in a photoreceptor degeneration model [14], and it is an inappropriate strategy in humans once irreversible loss of photoreceptors has occurred.

A protracted phase of inner neural retinal remodeling occurs after the depletion of the sensory photoreceptors; however, the initial stages largely involve remodeling of the outer nuclear layer [15,16]. Prior to the clinical application of any cell replacement strategy, the progress of global retinal remodeling should first be assessed in order to define the key impediments towards visual reconstitution. A sufficient retinal cyto-architecture must exist for successful graft integration to yield improvements in visual function. Functional cell replacement may be complicated by the retraction of interneuron dendrites and axon terminal fields, Müller glial and horizontal cell hypertrophy, and horizontal neurite sprouting towards the inner plexiform layer [15]. Thus the optimal period for cell engraftment within each disease paradigm should be determined. Nonetheless, a therapeutic window of opportunity for cell replacement may exist prior to significant inner retinal remodeling. Such strategies have proved successful in other degenerative regions of the central nervous system; for example, Parkinsonian symptoms have been reduced for up to six months in monkeys following chemical depletion of dopaminergic cells by transplanting monkey embryonic stem cells (ESCs) or ventral mesencephalon [17,18], and hind limb motor function can be improved in animal models of spinal cord injury following transplantation of olfactory ensheathing cells [19,20]. Cells grafted into the photoreceptor-depleted outer retina prior to the onset of global remodeling could potentially reconstitute the remaining retinal circuitry, partially restoring visual function.

Here we present the clinical manifestations and genetic correlations of a selection of hereditary macular dystrophies (HMDs) that may be suitable candidates for emerging cell- and gene-based therapy. The main focus of this review is the outer retina and diseases affecting central visual acuity where the primary defect results, either directly or indirectly, in the demise of photoreceptors. Here we concentrate on photoreceptor replacement rather than RPE replacement, although in many cases of HMD the replacement of both tissue types is required. We discuss current research successes, the potential implications and limitations of emerging techniques for visual restoration, potential combinatorial approaches, and possible future directions in this highly enigmatic field.

## GENETIC BASIS OF RETINAL DISEASE

### Hereditary Macular Dystrophy

Although modifiable lifestyle factors such as smoking and a high body mass index increase the risk of retinal disease, new evidence is emerging that stresses the importance of familial influence and the underlying molecular causes of retinal disease; indeed, few clinical conditions are totally without some genetic influence [21]. Below we describe three major types of HMD and discuss the genetic and environmental contribution to the onset and progression of retinal disease. A summary of other HRDs is presented in Table 1.

**Table 1:** A summary of the occurrence of cone and macular dystrophies and associated the identified underlying molecular causes

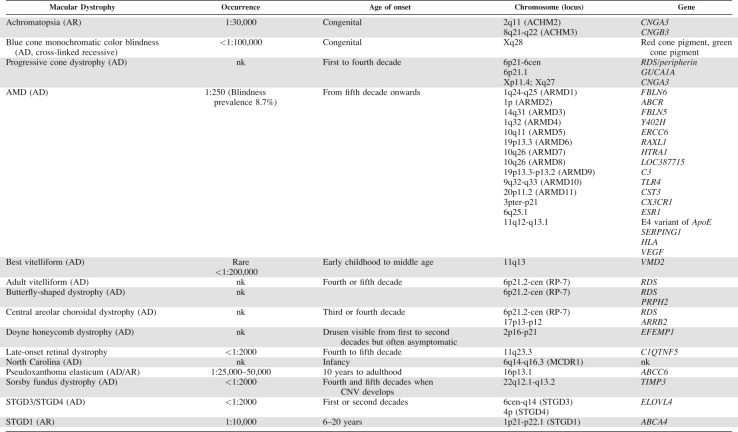

Abbreviations: AD, autosomal dominant; AMD, Age-Related Macular Degeneration; AR, autosomal recessive; CNV, choroidal neovascularization; nk, not known.

A feature of many HMDs, although typically characteristic of Age-Related Macular Degeneration (AMD), is the presence of sub-RPE deposits, or drusen [22–24]. This contributes to disease pathogenesis by cleaving RPE cell attachment to Bruch's membrane, which causes disruption of membrane transport and subsequent RPE demise [25]. Concomitant local oxidative stress, chronic inflammation, choriocapillaris changes, and neurosensory degeneration occur [26]. Various underlying molecular causes alter individual susceptibility for drusen accumulation and subsequent pathology in HMD; however, drusen and histopathological changes in RPE can also be detected during normal physiological aging [27]. Photoreceptors are continuously renewed by shedding outer segment (OS) distal disks in a circadian fashion [28]. With aging, the heavy metabolic burden of OS phagocytosis and breakdown on the RPE results in incomplete digestion of internalized material and accumulation of autofluorescent lipofuscin, a component of drusen, within RPE lysosomes and endosomes. A major fluorophore of RPE lipofuscin is A2E [29,30], a minor by-product of the visual cycle [30,31]. Given its high density of photoreceptors, the macula accumulates the greatest levels of A2E in its RPE. The RPE becomes less efficient in coping with increased toxic lysosomal A2E and other visual by-products, thus contributing to AMD [31]. Although fundoscopically similar, the age of onset, pattern of drusen deposits, and visual course separate normal aging from patients with HMD. AMD, once considered effectively an environmentally exacerbated form of aging, can now be considered another form of HMD with onset later in life.

#### STGD

STGD is the most prevalent HMD with an estimated incidence of one in 10,000 [32]. It accounts for 7% of all HRD and carries a 25% a priori risk to siblings [21]. The traditional presentation (STGD1) is autosomal recessive with juvenile onset (7-12 years); however, some rarer autosomal dominant forms exist [33–36]. STGD is clinically characterized by RPE and photoreceptor inner segment lipofuscin accumulation, RPE and choroidal vascular atrophy, macular photoreceptor loss, and reactive Müller glial hypertrophy [37,38] with progressive loss of central visual acuity during the first two decades [21]. *Fundus flavimaculatus*, characterized by subtly later onset, slower progression, and widespread, symmetrical, deeply localized retinal flecks [39,40] is understood to be a different manifestation of the same disease.

STGD dystrophies are mapped to chromosome 1p21-p22.1 and vary in onset, clinical course, and severity. This is likely due to allelic mutations, largely missense, producing a continuum of disease pathology and presentation [21,41,42]. The adenosine triphosphate–binding cassette (ABC) transporters function to aid adenosine triphosphate–dependent translocation of substrates across cell membranes and are implicated in human inherited disorders. Deletion of the ABC subfamily A (ABC1), member four (*ABCA4*, alternatively *ABCR*) gene [43,44], which encodes for the rod OS protein Rim (RmP) [45,46], functions in the transmembrane transport of vitamin A derivatives to the RPE and accounts for 60% of STGD cases [47]. Mutations in *ABCA4* have been identified in retinitis pigmentosa (RP) and cone-rod dystrophy [48–51]. *ABCA4* mutations are also associated with all juvenile HMD, recessive RP, and cone-rod degeneration [47,50].

*Abcr*^−/−^ mice display biochemical, physiological, and ultrastructural changes with delayed rod dark adaptation, delayed clearance of all-*trans*-retinaldehyde (all-*trans*-RAL), increased levels of PE in rod OS, accelerated A2E accumulation in the RPE, thickening of Bruch's membrane, and visual loss [52,53]. When raised in total darkness, A2E accumulation in *Abcr*^−/−^ mice is completely inhibited, indicating that the rate of STGD progression in humans might be slowed by limiting light exposure [54].

The two dominant forms are genetically distinct from recessive STGD, mapped to chromosome 6cen-q14 (STGD3) and 4p (STGD4). These affect the elongation of very long-chain fatty acid-like gene four (*ELOVL4*), expressed abundantly in photoreceptors [55]. A knock-in mouse model of a five base pair deletion in *ELOVL4* was recently reported [56]. Mice heterozygous for this mutation show photoreceptor degeneration, while the homozygous variety have abnormally compacted outer epithelium, lack key hydrophobic components of the stratum corneum affecting permeability barrier function, and die shortly after birth.

#### Sorsby Pseudoinflammatory Fundus Dystrophy

Sorsby pseudoinflammatory fundus dystrophy (SFD) is a highly penetrant, rare, autosomal dominant condition characterized by loss of central vision due to extracellular matrix abnormalities in Bruch's membrane and bilateral choroidal neovascularization (CNV). This disrupts choroidal nutrient and metabolite transport leading to atrophy of the neural retina [57,58]. Onset is typically 40-50 years of age [59,60]. Fine drusen-like deposits, atrophic lesions at the macula [21], and lipid deposits at the RPE/Bruch's membrane interface are observed [22]. This is usually complicated by CNV and associated hemorrhage leading to a disciform macular scar [61]. The peripheral retina is also affected, resulting in night blindness (nyctalopia).

SFD shares several features with late-stage AMD, resulting in SFD becoming an accepted AMD genetic model [62]. Whilst AMD has highly complex and largely unknown etiology, SFD is a single-gene disorder that occurs due to mutations in the *TIMP3* gene on chromosome 22 [63–65]. Its product is an RPE enzyme important in extracellular matrix regulation [66,67], and the presence of a mutant form may affect retinal protein turnover [61]. Eight mutations are described, seven affecting the coding sequence in exon 5; six are missense, introducing a novel unpaired cysteine residue into the C-terminal domain [61,62,66,68–70]. Other mutations introduce a stop codon at position 139 [71] or a single base (A) in the splice acceptor site between exons 4 and 5 (CAG to C*A*AG) [72]. A region of the *TIMP3* C-terminus appears particularly vulnerable; three mutations are found between residues 166-168 with two others (172 and 181) in close proximity [61]. The serine to cysteine substitution of residue 181 [Ser181Cys] in exon five is the causal mutation in the majority of Sorsby's families tested in the United Kingdom.

In SFD, mutant *TIMP3* accumulates in the RPE and Bruch's membrane, prompting the disease process [65,73,74]; *TIMP3* overexpression can induce apoptosis in several cell types including RPE [75]. Unlike other *TIMP* family members, *TIMP3* binds to the extracellular matrix via its C-terminal, the site of all known SFD mutations [61,76–78]. Whereas mice with targeted deletion of *TIMP1* show CNV [79], homozygous *TIMP3*-null mice do not exhibit an obvious retinal phenotype [80]. A knock-in mouse carrying a Ser156Cys mutation in the orthologous murine *TIMP3* gene shows clinical features of human SFD, including abnormalities and elevated TIMP3 in Bruch's membrane and RPE [68,81], providing an experimental system in which to investigate SFD pathophysiology. The range of mutations is limited in SFD, meaning that genetic analysis makes diagnosis quick and reliable, although the underlying disease process remains untreatable.

#### Age-Related Macular Degeneration

AMD is the leading cause of blindness in the developed world over 60 years of age and accounts for 50% of blind registration [82–87]. Incidence of AMD rises exponentially with age [88]. While its prevalence is increasing in the United Kingdom, blind registration from cataract, glaucoma, and optic atrophy has declined [89]. There are two main forms of AMD: an early form characterized by degenerative changes in the RPE and accumulation of drusen, and a late form that manifests with geographic atrophy alongside sub-RPE and subretinal CNV [90]. Therapies to prevent and treat AMD are limited. High-dose antioxidant vitamins and metabolic therapies can reduce the progressive visual loss in patients with early AMD and, in the case of metabolic therapy, the regression of drusen; however, the absolute reduction in new cases is small [91–93]. Although environmental factors are known to play a role in disease pathology and progression, epidemiological and family-based studies provide convincing evidence for a genetic basis for AMD, with inheritance thought to be polygenic [94–100]. Despite its prevalence, the etiology and pathogenesis of AMD remains poorly understood. Susceptibility loci (chromosome 1, 5, 9, 10, and 17) [101–103] and candidate genes that appear to play a role have been identified [104–108], some correlating with AMD pathology more strongly than others; more than 100 different proteins are associated with AMD deposits [108].

The complement system, involved in our immune defense against foreign antigens, is implicated in AMD [109–111]. Complement factor H (CFH) inhibits the complement cascade that ensures the system is directed against pathogens and not the body's own tissues, and is a major susceptibility gene in AMD. Complement components are found alongside drusen and the RPE-choroid interface [110], and abnormal macular dysregulation of the complement cascade in RPE and Bruch's membrane appears to cause unrestrained complement activation and drusen formation [112]. The CFH Y402H haplotype on chromosome 1q32 confers the greatest risk of AMD [113]; however, multiple polymorphism variants in caucasian AMD patients have been determined [104,114–118]. Y402H is not a primary indicator in Japanese patients, however [119], nor in the Chinese population in which this polymorphism is associated specifically with neovascular AMD [120]. The classical complement pathway is largely regulated by the SERPING1 gene product, and a common polymorphism in SERPING1 was recently identified as a causal factor in AMD [121]. Interestingly, a CFH haplotype with a deletion in CFHR1 and CFHR3 elicits an independent decreased risk of AMD [113].

Oxidative stress pathways are also causally involved [122]. Schmidt and colleagues identified a coding change [Ala69Ser] in the *LOC387715/ARMS2* gene on chromosome 10q26 as the second major identified susceptibility allele [123]. They report that genetic susceptibility in combination with a modifiable lifestyle factor conveys a significantly higher risk than either factor alone. The population-attributable risk is 36% for *LOC387715/ARMS2* and 43% for Y402H, however combining *LOC387715* and cigarette smoking increases risk to 61% [123]. The LOC387715/ARMS2 protein product colocalizes with the mitochondrial outer membrane in mammals [106]; thus, the Ala69Ser mutation presumably contributes to AMD pathogenesis by affecting mitochondrial function. Polymorphisms of other immune response genes, such as human leukocyte antigen, are also implicated after observation of strong human leukocyte antigen immunoreactivity in drusen [109,124]. Variants of complement cascade component factor B and complement component two are protective against AMD [105].

Several genes implicated in the etiology of various HMD have been examined in AMD. Carrier relatives of STGD are more likely to develop AMD [125], and genetic similarities may exist between the two [43]; however, it remains controversial as to whether the *ABCA4* gene plays a pathogenic role [43,44]. The high level of *ABCA4* polymorphism across individuals makes it difficult to determine any responsibility for *ABCA4* in AMD [21]; however, its involvement is possible in a small number of cases [107]. A2E, which accumulates in the STGD eye, is also found in the RPE in AMD and perturbs efflux of cholesterol from RPE endosomes/lysosomes, causing cholesterol and cholesteryl ester deposit accumulation [61,126,127]. Initially identified as a causal factor in Doyne's disease, a mutation in the extracellular matrix protein *Fibulin 3* gene [128] led to the correlation of missense mutations in *Fibulin 5* in 1.7% of 402 AMD cases in the United States [129]. These contribute to AMD by causing reduced Fibulin five and elastin production—a key Bruch's membrane component—but were not causal [129]. The *TIMP3* mutation in SFD has not correlated with AMD [66,130], although distribution of the *TIMP3* enzyme product in the AMD Bruch's membrane is significantly higher than age-matched controls, remains in active form, and is associated with drusen patches [76].

Late-onset retinal degeneration (LORD) is a rare autosomal dominant disorder that has striking parallels with AMD. Histopathology reveals thick sub-RPE deposits that result in RPE dysfunction and photoreceptor loss [24,131–133]. A Ser163Arg mutation in the *CTRP5*/*C1QTNF5* gene is reported to cause approximately 50% of documented LORD cases [134]. As yet it is unknown whether mutations in CTRP5 influence susceptibility to AMD. Apolipoprotein E (ApoE) and β-amyloid aggregates, associated with Alzheimer's disease, have been found in AMD-related macular drusen [135,136]. Some studies implicate the ApoE ɛ4 allele as a protective factor which delays disease onset, while the ɛ2 allele accelerates disease onset and progression [137,138]. However, although there is currently no significant association between Alzheimer's disease and AMD [139], a recent study supports some relationship between cognitive function and dementia with early AMD in older individuals [140]. Mutations in the *Bestrophin* gene, responsible for vitelliform macular dystrophy (Best's disease) show a small, nonsignificant correlation with AMD [141,142].

The identification of vascular endothelial growth factor (VEGF) polymorphisms in late-stage AMD has led to the use of optical coherence tomography-guided anti-VEGF (OCTVEGF) treatments which slow disease progression by treating CNV [143]. They do not, however, alter the underlying pathophysiology, and atrophic macular changes can still progress despite successful CNV treatment. A naturally occurring mouse model with autosomal recessive late-onset severe retinal degeneration mapped to the *Mdm1* gene on mouse chromosome 10 was recently reported, but is not associated with the human *Mdm1* ortholog or AMD [144]. The *Sod1*^−/−^ and ApoE knock-in mice show the most similarities to the human clinical manifestation of AMD thus serve as good disease models [145,146]. In conclusion, the most prominent genetic factors identified to date in the etiology of AMD are the Y402H variant of CFH, *LOC387715*, and *SERPING1*, which are found in more than 60% of cases [121].

### Cell Replacement Strategies

While modifiable lifestyle factors present an increased risk of retinal disease to an individual already expressing high-risk alleles, there is clear evidence that genetics plays an important role in the occurrence and development of HMD. Understanding the inheritance and genetic basis of these retinal diseases will undoubtedly provide new treatment platforms for restorative therapy. Gene therapy, whether corrective [147–150] or to overexpress various neuroprotective substances [135] either on its own or combined with stem cell therapy, has enormous potential in these diseases, although it may be less applicable to those cases where the dystrophy is advanced at birth or where perturbed retinal and cortical development has compromised visual function [151]. Stem cell therapy could be used in two main ways, namely by enhancing endogenous repair using stem cells that secrete growth factors or deliver drugs utilizing the innate attraction of diseased structures to various stem cell populations, or by exogenous cell replacement strategies. In the case of HMD, the latter strategy could be a useful therapeutic option depending on integrity of the inner retina, Bruch's membrane, and choriocapillaris, and provided that problems associated with the synaptic integration between transplanted photoreceptors and the host retina can be overcome.

Various sources of cells and methods of transplantation have been used in the attempt to replenish the retina with functional photoreceptors or healthy RPE following the degeneration of host cells in assorted animal models of retinal degeneration with various degrees of success [152–157]. In the best case, subretinal transplantation of embryonic and postnatal day 1 (P1) mouse retinal cells into murine models of retinal degeneration showed greatest integration, differentiation, and synaptic connectivity within host tissue when already committed to a photoreceptor fate while still morphologically immature. Increases in pupil sensitivity in this study correlated with the number of incorporated Nrl^+^ donor cells, a result that was not achieved with transplanted proliferating or stem cells [157].

Recent reports describe the successful generation of photoreceptor precursors from ESCs using defined protocols incorporating factors that are known to promote forebrain development, retinal progenitor specification and photoreceptor induction [158–161]. Similarly, protocols for RPE production from ESCs are being refined, and functional studies on human ESC-derived RPE demonstrate their physiological viability [162]. Small numbers of opsin- and rhodopsin-positive cells can be observed after subretinal transplantation of spontaneously differentiated human ESCs (hESCs) into neonatal and adult rat eyes with no tumor formation observed up to 18 weeks post-transplant [163]. More recently, retinal progenitors derived from hESC were subretinally transplanted into *Crx*^−/−^ mice, a model of Leber congenital amaurosis (LCA) [164]. Grafted cells integrated within the outer nuclear layer; displayed layer-specific expression of opsin, rhodopsin, and recoverin; and restored the light response in previously unresponsive recipients, even though grafted cells expressing photoreceptor-specific markers did not develop OSs. These results highlight the value of hESC as a potentially unlimited source of specialized cell types for transplantation; however, issues do remain regarding their use, including efficient direction of differentiation towards a required lineage and elimination of undifferentiated ESCs from the cell population intended for transplantation in order to minimize the risk of tumorigenesis.

The ETDRS chart, originally used in the Early Treatment of Diabetic Retinopathy Study, has become the standard distance visual acuity as measurement in clinical research [165]. More recently, modest improvements in visual acuity as measured using the ETDRS chart was reported after transplantation of fetal neural retina with its RPE in a patient with dominant RP [166]. These authors recently published their results from an additional fetal retina-RPE transplant clinical trial conducted on six RP and four AMD patients [167]. In this latest trial, 70% of transplant recipients showed improvements in visual acuity, and postoperative loss of RPE pigmentation was not associated with changes in visual outcome [167]. In one patient, improvement in visual acuity and light sensitivity was maintained for 6 years. It is difficult to discern, however, whether observed improvements are due to graft functionality, graft-derived factors, or surgical manipulation eliciting improvements in visual function [168,169]. The development of an artificial Bruch's membrane substitute or the use of scaffolds to maximize the adhesion and safe surgical delivery of the RPE sheets or tissue under the retina may enhance cell-replacement strategies [158,170] provided there is access to functional choriocapillaris. The absence of just one of these vital elements will cause a sequence of events affecting retinal viability and hinder graft integration and function.

In summary, although cell transplantation results are encouraging, only a small proportion of grafted cells (in the best case 0.4% of sorted P1 Nrl^+^ photoreceptor precursors) integrated within the correct lamina and differentiated appropriately in a model of retinal degeneration [158]; therefore, the efficiency of retinal reconstitution via cell replacement remains a challenge. Encouraging incorporation of grafted photoreceptor precursors is ineffective without a functional RPE/Bruch's membrane/choriocapillaris complex. Studies over the past 10 years have shown that allogeneic RPE largely resists attachment to aged Bruch's membrane in vitro and where successful attachment and proliferation has been achieved, long-term survival is poor [171–173]. Early studies in humans demonstrated that long-term survival of transplanted fetal retina with its RPE (up to 6 months) could be achieved in RP patients, but with no improvement in visual function [174]. Similar studies in AMD patients yielded mixed results; transplantation of fetal or adult RPE [175–178] implied that late-AMD is less conducive for graft survival with patients showing signs of rejection within 3 months [174]. RPE transplanted alongside aggregate retinal transplants or photoreceptor sheets into RP and AMD patients proved the long-term safety and survival of grafts but again patients showed no improvement in visual function [179–181].

### Revolutionary Developments in the Stem Cell Field: Induced Pluripotency

As highlighted in the above section, hESCs have been shown to differentiate along photoreceptor and RPE lineages using growth factors that have roles in forebrain development and early and late retinal fate specification. In one very recent study, hESC-derived photoreceptor cells were shown to settle into the appropriate layers and express markers of differentiated rod and cone cells upon intraocular injection into animal models of retinal disease [164]. These are undoubtedly promising results, but many substantial shortcomings of hESC differentiation still await resolution. Among these are the ethical issues associated with the use of embryonic tissue, the apparently embryonic/fetal phenotype of the cells derived during hESC differentiation [183], and the potential of tumorigenesis arising from the presence of undifferentiated progenitors remaining in culture; however, the principal problem is one of immune rejection of differentiated cells after transplantation into the patient. Pioneering work carried out in the last 3 years suggests that immune rejection issues may be overcome by creating embryonic-like stem cells from somatic cells of adult individuals through a process called induced pluripotency [184–186]. This process, which is held as one of the most seminal discoveries in the stem cell field, was first reported by Yamanaka's group [187] and involves overexpression of four key genes (*Oct4*, *Sox2*, *Klf4*, and *c-Myc*) in murine fibroblasts, resulting in their conversion into cells that resemble ESCs in terms of morphology, gene expression, growth, and differentiation capabilities and are now named induced pluripotent stem cells (iPSCs). This work was soon supplemented by the generation of human iPSCs by two groups, one of whom [186] showed that adult human dermal fibroblasts can also be reprogrammed by overexpression of *OCT4*, *SOX2*, *KLF4*, and *c-MYC*, while another [188] made use of a slightly different set of factors (*OCT4*, *SOX2*, *LIN28*, and *NANOG*) to reprogram both fetal and adult human fibroblasts. Since these initial papers there has been remarkable progress in the field, aimed largely at increasing the efficiency of the iPSC generation protocol and replacing the initial retroviral vectors that were used to transfect fibroblasts with *OCT4*, *SOX2*, *KLF4*, and *c-MYC*. Two approaches to this latter problem have been used with varying degrees of success. The use of nonintegrating retroviruses that maintain transient expression of the reprogramming factor genes from episomes that do not integrate into the host cell genome has shown some potential to generate iPSCs, albeit with lower efficiency than is achievable with integrating retroviruses [189]. Very recently, a protocol using recombinant proteins for induction of pluripotency has been published, which suggests that future methods for the production of iPSCs may be much simpler than those of the initial publications in this area [190].

Together these developments suggest that it is now possible to derive iPSCs free of transgenes and with perhaps reduced risk of tumorigenesis, which was observed in the initial animal-based studies (20–30%) associated with reactivation of *c-MYC* in adult tissues [191]. Caution is required, however, for the small molecules and chemicals that are required for reprogramming because they may promote global epigenetic and genetic modifications, which may compromise the safety aspect of these cells.

The ability to generate iPSCs readily allows us to progress to one of the principal applications envisaged for this technology, which is the generation of human disease models and potential correction of genetic disease. For example, iPSC technology was combined with gene therapy to correct the mutant human sickle cell anemia allele [192]. The corrected iPSC generated in this study were induced to differentiate to hematopoietic progenitors that were subsequently transplanted into the murine model of sickle cell anemia, leading to functional recovery and providing a “proof of principle” demonstration for future human therapeutic application. Similarly, transplantation of murine iPSC-derived neural progenitors into animal models of Parkinson's disease led to improved behavior 4 weeks after transplantation [193]. Furthermore, iPSCs have been generated from other species, including the rhesus macaque, which is by far one of the most relevant primate models for human disease [194].

To date, a large number of iPSC lines from various diseases such as amyotrophic lateral sclerosis [195]; Parkinson's disease [196]; type I diabetes [197]; spinal muscular atrophy [198]; adenosine deaminase deficiency-related severe combined immunodeficiency (ADA-SCID); Shwachman-Bodian-Diamond syndrome; Gaucher disease; and type III, Duchenne, and Becker muscular dystrophy [199] have been reported.

Creation of iPSC lines on its own is not sufficient and needs to be combined with techniques that have and will be developed in human ESC/iPSC for efficient and directed differentiation towards the desired functional cell type. In view of this, it is important to determine whether iPSCs differentiate into various phenotypes in a manner similar to ESCs, and, although a couple of studies have suggested that this may be the case under in vitro conditions [200–202], in vivo functional studies in animal models need to be performed to fully investigate the potential of iPSC lines for both disease modeling and cell transplantation.

### Potential Applications and Limitations of iPSCs for Understanding and Treatment of Retinal Disease

Derivation of iPSCs from patients with diseases causing outer retinal degeneration, alongside in vitro gene correction with a robust differentiation method for producing photoreceptor and RPE cells from these cells, could provide a source of autologous cells for transplantation. A very recent report has shown that human iPSC can be differentiated to photoreceptor cells with efficiency similar to that of human ESCs [203], suggesting that already established protocols for human ESCs can be transferred directly to iPSCs. Of equal importance is the derivation of iPSCs from patients with retinal disease for creation of disease models, which provides an invaluable opportunity to investigate disease pathogenesis and treatment that has not been possible before. Although there are successful animal models of retinal degeneration, most of these models mimic modulation of one or at most two genes [204]. Using iPSCs isolated from affected patients provides new opportunities to complete the current gaps in our understanding [205], especially for disorders such as AMD where multiple genes are likely to play a role in disease initiation and progression as highlighted above (see Age-Related Macular Degeneration) and where animal models that mimic the disease perfectly are difficult to create due the polygenic nature of the disease. Such cells can be used to devise genetic tests for risk prediction and diagnosis as well as testing and design of new drugs that may have an impact on photoreceptor and RPE cell degeneration.

Generation of photoreceptor and RPE cells from patient-specific iPSC lines overcomes the need for immunosuppression and will likely reduce the risk of rejection of grafted tissue within the neurally depleted region. However, in diseases where a single underlying genetic mutation is causing cellular dysfunction or death, one caveat in generating iPSCs from such patients will be the generation of populations of cells that carry the same mutation. Targeting host photoreceptors with gene therapy to supply growth factors or antiapoptotic genes has already been tested in animal models of degenerative retinal disease in addition to antiangiogenic factors in animal models of neovascular retinal disease, with no adverse morphological, inflammatory, or vascular effects [11]. It is therefore possible that the experience gained from gene therapy trials in adult host photoreceptors can be applied to iPSC-derived photoreceptor or RPE cells. Another possibility is the correction of the gene defect at the iPSC stage prior to differentiation to photoreceptors or RPE, although the polygenic nature of many HMD cases means that such an approach currently remains a challenge. Nonetheless, although homologous recombination is still very difficult in hESCs and presumably in iPSCs, inducing the expression of the wild-type gene in mutant iPSCs prior to their differentiation is likely to be achieved using viral vectors, which fits with recent reports of gene correction [148–150]. For example, a recent report has shown that subretinal lentiviral vector delivery of the human *ABCA4* gene in a mouse model of STGD was shown to transduce up to 20% of photoreceptors in the injected region and substantially reduce disease-associated lipofuscin accumulation [206]. It can be therefore envisaged that expression of wild-type *ABCA4* in iPSCs derived from well-characterized STGD patients with *ABCA4* mutations and their further differentiation to RPE and photoreceptor cells could potentially elicit some clinical improvement. Conversely, in cases where differing clinical manifestations of HRD arise from the same gene mutation (for example, adult vitelliform, central areolar choroidal, and butterfly-shaped dystrophies all arise from a mutation in the *RDS* gene on the short arm of chromosome six (Table 1)), the same corrective gene therapy might be applied to patients spanning more than one dystrophy, eliciting wider clinical impact. Care should be exercised with this approach, however, because overexpression of particular genes may carry the additional risk of unwanted and unpredicted physiological effects. In addition, applications of this approach for other single-gene disorders will depend very much on the size of the gene of interest because vectors have limited packing capacity, which may convey limitations in terms of the size of the defect that could be fixed. This approach will not work in genetic diseases associated with dominant negative effect such as STGD patients with *EL0VL4* mutations or SFD patients with *TIMP3* [207,208]. In these cases, gene correction at the iPSC stage is likely to be the only means of gene correction. There are already developments in this field mainly applied to hESCs using zinc finger nucleases [209]; however, this method has to be established and tested in human iPSCs and in multiple loci with high efficiency before this approach can be envisaged as a tool in cell and gene therapy.

Correction of gene defects in other retinal disorders such as AMD already associated with multiple genes, polymorphisms, and environmental factors, however, is unlikely using the iPSC approach, and in this case the usefulness of iPSC technology will mainly rely on the development of screening tools that will enable identification of individuals with high-risk of advanced AMD prior to retinal degeneration.

### Translation: Challenges Ahead?

Efficient tissue delivery, graft integration, and synaptic connection with host circuitry remain as issues for the successful clinical translation of cell-based restorative therapies [210]. In patient terms, those affected by severe macular degeneration may be more willing to consider the option of translational human surgery. There is rarely any HRD-associated cognitive impairment, which means that informed consent could be performed with the patients prior to any translational research. Exploiting HMD with well characterized causal mutations is an excellent starting point for the translation of gene therapy combined with iPSC cell-replacement strategies from bench to bedside. For example, STGD is relatively well characterized and quite common, making it important and clinically relevant, and it primarily affects the macula, thus allowing potentially simple surgical replacement of macular cells with genetically-corrected iPSCs.

Cell replacement requires the stable and appropriate nondisruptive integration of functional cells within the correct lamina, the formation of synapses with appropriate interneurons, and the correct electrophysiological and transmitter responses to light to enable the transmission of discernable visual responses to the cortex. It also requires the long-term survival and viability of grafted cells. Variables in the transplantation procedure in humans include the source of cells, age of the donor, transplantation method, and stage of host disease progression [211,212]. The presence of xenogeneic molecules in protocols used to direct derivation and differentiation in vitro will limit clinical use. Similar to the use of hESCs, driving patient-specific stem cells towards a desired lineage in appropriate numbers remains a challenge. Recent advances in differentiating stem cells to photoreceptors have been achieved, meaning there are already some methodologies in place for generating the cells of interest [160,161,213], and the results of the study by MacLaren et al. provides information about the developmental time point to which we should drive cellular differentiation to encourage graft integration [158]. Although much work needs to be done to enhance the incorporation of grafted cells, it may be that in some patients only a small number of integrated cells are needed for functional restoration. In one study, the onset of obvious visual symptoms in glaucoma patients was reported only after loss of 25-35% of ganglion cells [214]. It should be noted here that the possibility of correcting the underlying etiology in the end stage of disease by iPSCs, gene correction, and transplantation is attractive, but the comprehensiveness of this approach is dependent on the viability of other vital supportive retinal components. Patients affected by perturbed retinal cytoarchitecture will be less amenable to this therapy, making clinical assessment of these parameters by in vivo imaging and electrophysiology vital.

The continued identification of candidate genes and suitable delivery vehicles is key. Research into the molecular genetics of HRD is at an early stage, making disease classification on the basis of molecular pathology difficult. Patients previously have been divided by phenotype; however, the importance of accurately genotyping individual cases must be realized. This is likely currently complicated by expense or the lack of a well identified gene defect with an available test for exploitation.

Additionally, HRDs are inherited due to numerous types of mutations (loss of function, gain of function) and are clinically and genetically heterogeneous [215]. Mutation screening in STGD patients has led to the identification of 400 sequence variations in the *ABCA4* gene [216–219]. In early-onset HRD, genetic tests are able to be conducted more readily; however, this becomes problematic in the case of AMD where, due to the late onset, parents of affected individuals are often deceased and their offspring yet to be affected [220]. In representative animal models of HRD, the efficiency of gene correction techniques can be tested [53,81], and this will be further enhanced by the availability of patient-derived iPSCs.

One concern might be whether inheritance of HRD will affect gene therapy potential; for example, STGD is recessive and the mutations give variable penetration and expressivity. However, recessive models have been more amenable to gene therapy by restoration of the wild-type form of mutant genes. Photoreceptor rescue was achieved in 1996 by Bennett and colleagues who used adenovirus to introduce wild-type PDE6β in the *rd* mouse [221], an effect that was improved by the use of second-generation adenovirus and then lentivirus [222,223]. The results of three independent human retinal RPE65-replacement trials, published recently, demonstrate the safety of subretinal vector delivery in humans and show that gene therapy in advanced cases of human LCA could induce modest improvements in vision [148–150,224]. Two of the studies [148,149] used the ETDRS chart and an obstacle course combined with various other tests; however, only one reported electroretinography results [149]. Each study utilized recombinant AAV-2 vector delivered subretinally. In the study by Maguire et al. in Philadelphia [148], human *RPE65* carrying a chicken β-actin promoter was introduced into three patients, two with homozygous missense and one with null mutation in RPE65. Patients reported improvements in vision at 2 weeks and measured improvements in pupillometry and nystagmus frequency over several weeks, concomitant with improvements in confidence and time taken to complete an obstacle course in all subjects [148]. The London study reported by Bainbridge et al., [149] delivered the human *RPE65* gene under a human *RPE65* promoter in three LCA patients with RPE65 missense mutations. Unlike the first study, only one patient showed visual improvement as measured by ETDRS alongside significantly improved retinal sensitivity, dark-adapted perimetry, and dramatic improvements in mobility through an obstacle course (from 77 to 14 seconds). Electroretinography results however, were not improved [149]. The third study reported treatment of three young patients with LCA. All three patients showed significantly increased visual sensitivity in vector treated retinal regions, yet the kinetics of the newly restored retinoid cycle in rod and cone photoreceptors was slow [150]. In all studies there were no adverse systemic effects. The safety of the technique has thus been demonstrated, though visual improvements were modest, which suggests that perhaps treatment needs to occur at an earlier stage in the disease course or in combination with cell-replacement therapy.

It is important to show that iPSCs can be generated from older patients and whether patients at later stages of disease are amenable to combinatorial therapy. Bainbridge et al. proposed that the patient who benefited from gene therapy did so due to being treated while at a less advanced stage than their counterparts [149], highlighting the importance of understanding the optimal window of opportunity for genetic intervention. It cannot be ruled out, however, that observed differences between the studies may have occurred due to the use of different promoters, the subretinal delivery of varying amounts (150 μl vs. 1,000 μl), or slight variations in methodology.

Furthermore, although the eye and subretinal space in particular is relatively immunologically privileged, the immune response may be exacerbated and affect graft survival in HRD cases where the blood retinal barrier is compromised [11,211]. There is evidence that AMD has an inflammatory element, and avoiding any element of immune rejection would be vital in terms of disease recurrence. Use of iPSCs would avoid an immune response to the donor cells themselves, but genetically engineered modifications are being made to viral vectors to limit the immune response using this approach [225].

### Questions and Future Directions

Whilst developing the potential of iPSCs as a therapeutic strategy overcomes many of the issues associated with the use of hESCs (ethics, immunorejection), there are some very important control experiments that must be performed before translation of iPSC-based cell therapies to the clinic can become a reality. Fundamental questions include: Are these cells identical to hESCs? Do they have a normal karyotype? Can we efficiently differentiate iPSCs to photoreceptors and RPE? Given recent successes in generating photoreceptor precursors and functional RPE from hESCs, one would imagine similar results could be achieved with iPSCs. There is already evidence that iPSCs can differentiate into derivatives of the three germ layers [186]; however, it remains unknown whether retroviral transduction has any long lasting effects or whether cells might be reactivated under stress or with a lack in instructive cues, leading to the presence of potentially oncogenic cells. In light of this last comment, virus-free, nonintegrating plasmid reprogramming has been demonstrated in embryonic mouse fibroblasts [226], albeit at low efficiency compared with retroviral methods. These nonviral iPSCs were free from transgene integration in host chromosomes, demonstrated a capacity to differentiate towards all three germ layers, and could produce chimeric mice when injected into blastocysts. It is clear that while HMD with clearly identified etiology and a common causal mutation are the best candidates for combinatorial iPSC/gene/transplantation therapy, much work remains to be done to transform the current course of action after diagnosis of HMD for such therapy to become a reality in the near future. Improved knowledge of the mechanisms and molecular basis of HMD alongside continued safety evaluation of the long-term action and consequences of gene therapy and cell restoration will allow us to move towards tailoring safe, more specific and efficient restorative therapies for patients affected by this heterogeneous group of currently incurable diseases.
